# An mRNA vaccine with broad-spectrum neutralizing protection against Omicron variant sublineages BA.4/5 -included SARS-CoV-2

**DOI:** 10.1038/s41392-022-01207-4

**Published:** 2022-10-11

**Authors:** Ye Sang, Zhen Zhang, Entao Li, Haitao Lu, Jinrong Long, Yiming Cao, Changxiao Yu, Tiecheng Wang, Jing Yang, Shengqi Wang

**Affiliations:** 1 Beijing Institute of Microbiology and Epidemiology, Beijing, 100850 China; 2 School of Life Science, University of Hebei, Baoding, 071002 China; 3 Veterinary Research Institute, Chinese Academy of Agricultural Sciences, Changchun, 130000 China

**Keywords:** Nucleic-acid therapeutics, Gene therapy

**Dear Editor**,

The COVID-19 pandemic, caused by SARS-CoV-2, has resulted in more than 500 million confirmed cases, with over 6 million deaths by June 2022. Since the outbreak of the pandemic, five variants, including Alpha, Beta, Gamma, Delta, and Omicron, have been classified as VOCs^1^. As a result of selection pressure and continued replication of the virus in human populations, new variants are sure to emerge, which will affect the spread of SARS-CoV-2 and vaccine efficacy^1^.

Accumulated evidence demonstrated that two-dose Wild type (WT)-based mRNA vaccines effectively induce neutralizing immunity to Delta and Beta, albeit with varying degrees of decline, but had low or completely absent neutralizing antibodies against Omicron^2^. Therefore, it is imperative to develop an mRNA vaccine against Omicron because of its high infection rate and immune evasion properties. Indeed, a recent study found that Omicron-based mRNA vaccines induced potent neutralizing antibodies against Omicron but could not resist other SARS-CoV-2 variants^3^. In this study, we prepared and identified a Delta RBD-based mRNA vaccine with broad-spectrum neutralization against SARS-CoV-2 WT, Beta, Delta and Omicron variants. We designed 4 Delta RBD sequences with different UTRs and codon optimizations using Delta’s RBD as the target antigen for the mRNA coding sequence (CDS) (Fig. 1a). The most efficient of these mRNA constructs, mRNA-D2, was selected by Western blotting based on its in vitro expression levels (Fig. 1b). Western blotting results confirmed that Delta-RBD could be expressed in multiple cell lines (HEK293T, RD, and Huh7) in vitro (supplementary Fig. 1).

**Fig. 1:**
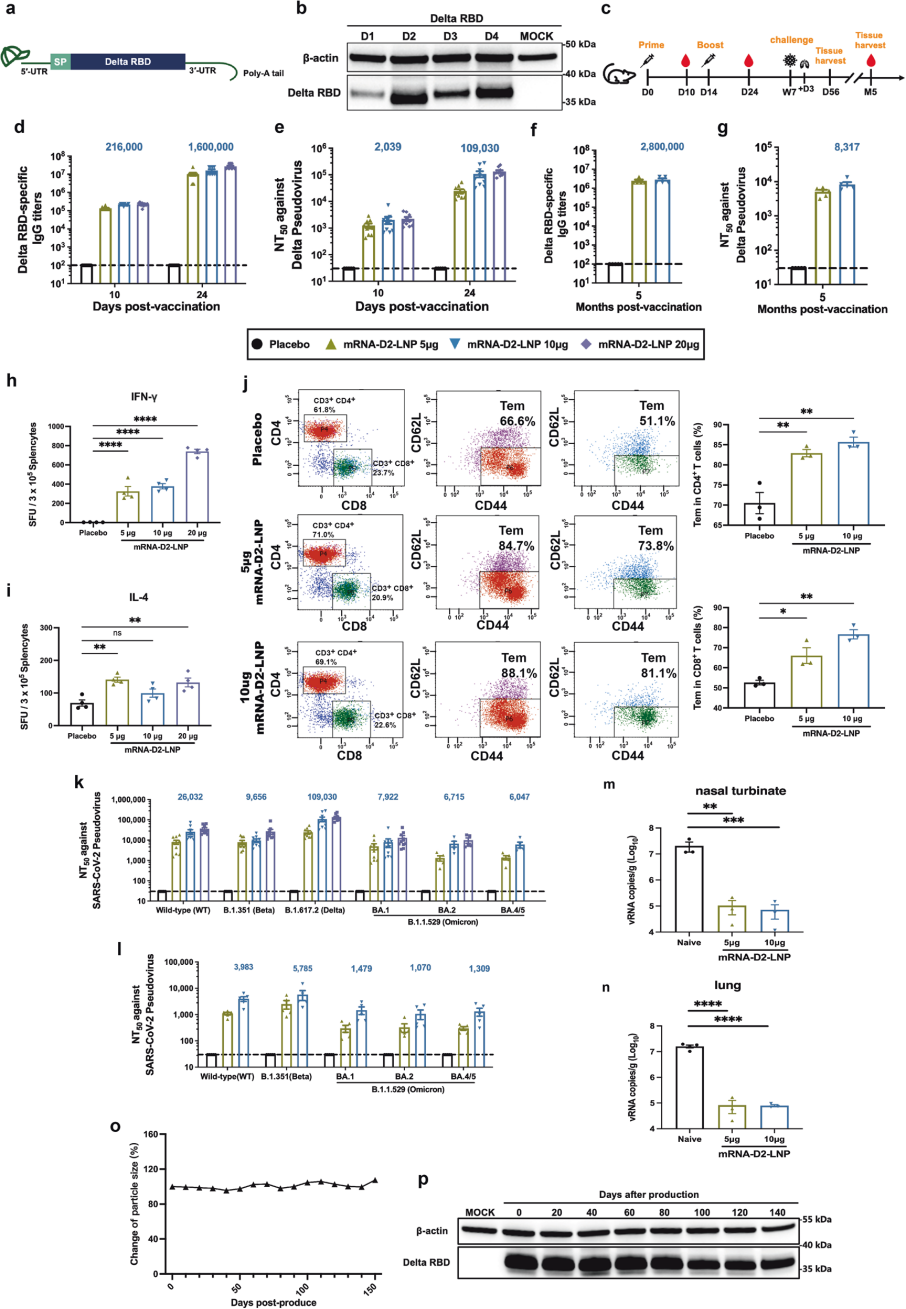
Development and characterization of an mRNA vaccine with broad-spectrum neutralizing protection against SARS-CoV-2 Omicron. **a** The mRNA construct of mRNA-D2-LNP expressing the SARS-CoV-2 RBD. **b** Delta RBD protein is expressed by mRNA in HEK293T cells. Cells were individually transfected with 4 mRNAs encoding Delta RBD (1 μg/mL) using Lipofectamine 2000 transfection reagent, and lysates were detected by Western blotting 16 hours after transfection. **c** Schematic diagram of mRNA-D2-LNP immunization, sample collection, and immunological assays. d, days; w, weeks; m, months. At days 10 and 24, Sera from immunized mice were assessed for Delta RBD-specific IgG antibodies (**d**) and pseudovirus-neutralizing antibodies (**e**) against Delta. After 5 months, Sera from immunized mice were assessed for Delta RBD-specific IgG antibodies (**f**) and pseudovirus-neutralizing antibodies (**g**) against Delta. ELISpot detection of IFN-γ (**h**) and IL-4 (**i**) release from SARS-CoV-2 RBD peptide stimulated splenocytes 56 days after mRNA-D2-LNP immunization. SFU = spot forming units. **j** Detection of SARS-CoV-2 RBD-specific CD4^+^ and CD8^+^ Tem cells (CD44^+^CD62L^-^) in splenocytes 5 months after mRNA-D2-LNP immunization by flow cytometry. **k** At day 24, Sera from immunized mice were assessed for pseudovirus-neutralizing antibodies against WT, Beta, Delta, BA.1, BA.2, and BA.4/5. **l** After 5 months, Sera from immunized mice were assessed for pseudovirus-neutralizing antibodies against WT, Beta, BA.1, BA.2, and BA.4/5. The mice were challenged intranasally with Omicron BA.1 (3.0 × 10^3^ TCID_50_) at week 7 after immunization. Nasal turbinate (**m**) and lung (**n**) samples were collected 3 days after challenge to assess viral RNA load by RT-qPCR. **o** Stored at 4 °C mRNA-D2-LNP was measured particle size and PDI every ten days and particle size change were analyzed by GraphPad Prism 8.0. **p** The mRNA-D2-LNP was transfected in 293 T cells every 20 days (mRNA-D2 = 1 μg/ml) and the lysates were collected for Western blotting to detect the change in Delta RBD protein expression. The numbers are the mean of the corresponding groups. Data were analyzed by one-way ANOVA with multiple comparison tests. (no significant (ns) *p* > 0.05, *p < 0.05, **p < 0.01, ****p < 0.0001)

After two doses of intramuscular immunizations at the 14-day interval (Fig. 1c), mRNA-D2-LNP induced potent Delta-specific IgG antibodies and neutralizing antibodies 24 days after initial immunization (Fig. 1d, e). We set up three groups of 5 μg, 10 μg, and 20 μg, and the antibody titers induced by mRNA-D2-LNP exhibited dose-dependent properties. To explore whether mRNA-D2-LNP conferred durable protection in immunized mice. We established a 5-month antibody follow-up experiment and observed that sera collected from mice still exhibited potent Delta-specific neutralizing antibodies after 5 months (Fig. 1f, g).

Then, we investigated the ability of two doses of mRNA-D2-LNP to trigger T cell immunity against SARS-CoV-2. Effective germinal center (GC) reactions are fundamental for the induction of high-quality SARS-CoV-2 neutralizing antibodies^4^, and T follicular helper (Tfh) cells are critical regulators of GC responses. In contrast to Tfh cells from S mRNA vaccine-immunized mice, which showed a more pronounced trend toward Th1 polarization, Tfh cells induced by the RBD mRNA vaccine had a mixed Th1-Th2 functional profile, capable of producing IFN-γ (Th1) and IL-4 (Th2)^4^. This is consistent with our study, where ELISpot assays showed significant IFN-γ and insignificant IL-4 production following mRNA-D2-LNP immunization (Fig. 1h, i). Even though more IFN-γ was produced in the test, this may be because the vaccine resulted in a CD4 T-cell response, which is mainly driven by Th1. It may be possible to avoid vaccine-associated enhanced respiratory disease (VAERD) by using mRNA vaccines to promote Th1/Th2 Tfh cells and conventional CD4 T cells with a Th1 bias. We further assessed the ability to induce CD4^+^ and CD8^+^ effector memory T cells (Tem) in immunized mice after 5 months. Flow cytometry results showed that mRNA-D2-LNP generated a long-lasting T cell memory effect (Fig. 1j).

To further determine the cross-neutralization of mRNA-D2-LNP, we measured the titer levels of multiple pseudovirus-neutralizing antibodies and conducted animal challenge experiments. In our study, mice booster immunized by mRNA-D2-LNP produced potent cross-neutralizing antibodies. 10 μg of mRNA-D2-LNP produced NT_50_ against WT, Beta, Delta, BA.1, BA.2, and BA.4/5 of 1/26,032, 1/9,656, 1/109,030, 1/ 7,922, 1/6,715 and 1/6,047 (Fig. 1k). Although several studies have reported that receiving the third dose of an mRNA-based vaccine produces a cross-neutralization response against Omicron BA.1^2^. It has been argued that the so-called “effective cross-neutralization response” is the result of “affinity maturation”, over time, producing sterilizing immunity based on high affinity for conserved epitopes among variant pathogens^2^. BA.4 and BA.5, which carry the same spike protein, have become the dominant strains worldwide. However, BA.4/5 has proven resistant to booster vaccines and immunizations caused by Omicron natural infections^5^. Remarkably, two doses of mRNA-D2-LNP immunized mice produced high titers of cross-neutralization antibodies against the Omicron variant sublineages BA.1, BA.2, and BA.4/5 with NT_50_ of 1/ 7,922, 1/6,715 and 1/6,047, respectively. In contrast, the WT-based mRNA vaccine was substantially undetectable for neutralization against BA.1, BA.2, and BA.4/5 (supplementary Fig. 2), consistent with reports from two marketed WT-based mRNA vaccines (mRNA-1273 and BNT162b) that neutralization of Omicron was essentially undetectable^2^. mRNA-D2-LNP induced high levels of neutralizing antibodies against four subvariants of Omicron. It also confirms that our vaccine might causes potent neutralization even against the latest variants. And we measured the level of cross-neutralization 5 months after immunization. Although the neutralizing antibody was significantly reduced after 5 months compared to the peak neutralizing antibody, it was detectable in each mouse against a different variant (Fig. 1l).

Afterwards, to further assess in vivo protective efficacy, mice immunized with two doses of mRNA-D2-LNP were challenged intranasally with Omicron BA.1 (3.0 × 10^3^ TCID_50_) at 7 weeks post-immunization. On day 3 post-challenge, the viral RNA levels were assessed in lung and turbinate tissues, and histopathological tests were performed. In contrast to those injected with DPBS, mRNA-D2-LNP provided >2 log reductions in viral RNA copies per g in nasal turbinate and lung (Fig. 1m, n). Moreover, more extensive lung lesions were found in DPBS-injected mice, with more lung lobes showing epithelial tissue degeneration, alveolar septal thickening, and activated inflammatory cells (supplementary Fig. 3). In contrast, lung lesions were largely not observed in vaccinated animals. These results demonstrated that vaccination with mRNA-D2-LNP prevented BA.1 replication in the upper and lower respiratory tracts and protected mice from lung lesions.

Additionally, we characterized the in vivo expression patterns of this mRNA-LNP formulation. After intramuscular injection, it was observed that strong protein expression was easily detected at the injection site (supplementary Fig. 4), which showed similar results to other LNP formulations^3,4^. Our data from mice then showed that 5 μg of mRNA-D2-LNP was sufficient to induce high-level neutralizing antibodies. We thus evaluated the safety of 20 μg of mRNA-D2-LNP in a mouse model. Biochemical parameters and pathological analysis indicated that even the highest dose did not cause significant adverse effects, which significantly improves the safety of our mRNA-LNP formulation (supplementary Fig. 5). Afterward, we assessed the thermostability of mRNA-D2-LNP. Upon storage at 4 °C for up to 150 days, long-term stability testing was conducted by monitoring particle size and in vitro protein expression. The results revealed that particle size remained constant over time (Fig. 1o), and expression efficiency remained approximately 100% up to 80 days and 80% up to 140 days (Fig. 1p and supplementary Fig. 6).

In summary, we report an mRNA vaccine with cross-neutralization protection against SARS-CoV-2 WT, Beta, Delta, BA.1, BA.2, and BA.4/5 based on Delta RBD. As a result of its enduring stability and robust safety, mRNA-D2-LNP is expected to be the next generation of mRNA vaccine for COVID-19. As anticipated for single-stranded RNA viruses, the genome of SARS-CoV-2 undergoes random mutations over time. With the emergence of the next generation of VOCs inevitable, mRNA-D2-LNP is expected to protect against infection-related symptoms, hospitalization, and death.

## Supplementary information

Supplementary Materials and Methods; Supplementary Figures S1-S6

## Data Availability

The data used to support the findings of this study are available from the corresponding author on reasonable request.
