# Supplementary material for: An mRNA vaccine with broad-spectrum neutralizing protection against Omicron variant sublineages BA.4/5 -included SARS-CoV-2
Source: Signal Transduct Target Ther. 2022 Oct 11;7:362. doi: 10.1038/s41392-022-01207-4 (PMC9552729; doi:10.1038/s41392-022-01207-4)
Supplement: Supplementary file 1 — Supplementary Materials and Methods; Supplementary Figures S1-S6 [file 41392_2022_1207_MOESM1_ESM.docx]

**Supplementary Materials for**

An mRNA vaccine with broad-spectrum neutralizing protection against Omicron variant sublineages BA.4/5 -included SARS-CoV-2.

Ye Sang, Zhen Zhang, Entao Li, Haitao Lu, Jinrong Long, Yiming Cao, Changxiao Yu, Tiecheng Wang, Jing Yang, Shengqi Wang

Correspondence: [sqwang@bmi.ac.cn](mailto:sqwang@bmi.ac.cn); [jingyang0511@sina.com](mailto:jingyang0511@sina.com); [wgcha@163.com](mailto:wgcha@163.com)

**This PDF file includes:**

Supplementary Materials and Methods

Supplementary Figures 1-6

**Materials and methods:**

**Cells and animals**

HEK293T (ATCC, CRL-3216), Huh7 (Preserved by our laboratory) and RD (Preserved by our laboratory) were cultured in Dulbecco’s Modified Eagle Medium (DMEM; Thermo Fisher Scientific) supplemented with 10% fetal bovine serum (FBS; Thermo Fisher Scientific) and penicillin (100 U/ml)-streptomycin (100 mg/ml) (Thermo Fisher Scientific).

Mice (female, BALB/c, 6-8 weeks of age, SPF) were obtained from Beijing Vital River Laboratory Animal Technology Co., Ltd. (Beijing, China). All animals were housed and bred in the temperature-; humidity- and light cycle-controlled SPF mouse facilities (20 ±2 °C; 50 ±10%; light, 7:00–19:00; dark, 19:00–7:00) in AMMS.

**mRNA design**

The Delta mRNA molecule began with a 5’-Cap 1 and incorporated deferent 5’-and 3’-untranslated regions (UTRs), then ended up with a 120 nt-Poly-A tail. The coding sequence region starting with AUG encoded the signal sequence (residues 1-14) and RBD (residues 319-541) of the spike glycoprotein genes of the SARS-CoV-2 Delta isolate.

**mRNA preparation and characterization**

All optimized mRNA sequences encoding Delta RBD was prepared by *in vitro* transcription using the T7-FlashScribe™ Transcription kit (Cellscript) from a linearized DNA template which incorporated 5’untranslated regions (UTR), signal peptide sequences, coding sequence (CDS), 3’UTR, and a poly-A tail were constructed. The transcription was followed by enzymatic capping of the RNA using ScriptCap™ Cap 1 Capping System kit with ScriptCap™ Capping Enzyme and 2'-O-Methyltransferase (Cellscript) according to manufacturer's instructions. Then, the mRNA product was purified by ammonium acetate precipitation was resuspended in RNase-Free water for further analysis and application. By using Agilent 2100 Bioanalyzer and RNA Nano 6000 Assay Kit (Agilent), the concentration and quality of the synthesized mRNA-D2 were measured. TriLink Bio Technologies provided the mRNA products encoding firefly luciferase (FLuc).

All Delta RBD-encoding mRNAs (1 μg) were transfected into HEK293T cells using Lipofectamine 3000 Transfection Reagent (Thermo Fisher) following the manufacturer’s instruction, and the lysates were collected and subjected to Western blotting.

**Preparation of mRNA-LNP**

Lipid-nanoparticle (LNP) formulations were prepared using a ultrafluidics of INano™L (Micro Nano Technology Inc). In brief, a lipid containing an ionizing lipid, 1, 2-distearoyl-sn-glycero-3-phosphocholine (DSPC), cholesterol, and DMG-PEG2000 was dissolved in ethanol (with molar ratios of 50:10:38.5:1.5). In a T-mixer at INano™L, the lipid mixture was combined with 20 mM citrate buffer (pH 4.0) containing mRNA at a 1:3 volume ratio. Formulations were then diafiltrated against 10 x volume of DPBS (pH 7.4) through a 100 k MWCO PES membrane (Sartorius Stedim Biotech) and concentrated to desired concentrations. The size and zeta potential of the mRNA-LNP were measured by a Litesizer 500 (Anton Paar) and data were analyzed using an Anton Paar Kalliope software package.

**mRNA transfection**

HEK293T, Huh7 or RD cells were seeded in 12-well plates at 300,000 cells/well. After transfection of the cells with mRNA-D2-LNP (1 μg/ml) for 16 hours, we collected the lysates for western blotting to test the *in vitro* delivery capability of mRNA-D2-LNP. The Delta RBD protein was then detected by western blotting with a poly-clonal antibody (pAb) against the SARS-CoV-2 RBD protein (Sino Biological).

**BLI for validation of the *in vivo* delivery of mRNA-FLuc-LNP**

To test the delivery capability of mRNA-LNP *in vivo*, mRNA-FLuc was encapsulated with LNP. Female BALB/c mice (6-8 weeks old) were administered 10 μg of mRNA-FLuc-LNP or DPBS (negative control) via intramuscular injection (n = 3). After 6 h post inoculation, mice were injected intraperitoneally with luciferase substrate (PerkinElmer). Following the reaction for 3 minutes, bioluminescence signals were collected by an IVIS Spectrum instrument (PerkinElmer) for 60 s. The bioluminescence signals in regions of interest (ROIs) were quantified using Living Image 3.5.

**Mouse vaccination** **and challenge experiments**

In this study, female BALB/c mice (6-8 weeks old) were intramuscularly vaccinated with mRNA-D2-LNP (5 μg, 10 μg, and 20 μg; n = 10) or DPBS at day 0 and 14. Sera were collected at 10 days, 24 days, 5 months and 8 months after initial immunization for detection of IgG antibody and neutralizing antibodies as described below. After 56 days and 5 months post initial immunization, splenocytes were collected for enzyme linked immunospot analysis (ELISpot) and flow cytometry as demonstrated below.

The SARS-CoV-2 challenge model based on Omicron BA.1 variant. BALB/c mice immunized with mRNA-D2-LNP were challenged intranasally with BA.1 (3.0×10^3^ TCID_50_) at 7 weeks post-immunization. On day 3 post challenge, all animals were euthanized, and the lung and nasal turbinate tissues as well as sera were collected for viral RNA level determination, histopathology assay as described below.

**Evaluation of serum antibody**

Delta RBD specific IgG antibody titers were determined by enzyme linked immunosorbent assay (ELISA). NT_50_ against WT, Beta, Delta, and Omicron were determined by a pseudovirus-based neutralization assay.

1. ELISA assay.

Delta RBD specific IgG antibody titers were determined by ELISA assay. 96-well plates were coated with 2 μg/ml SARS-CoV-2 Delta RBD protein (40592-V08H90, Sino Biological) and were incubated at 4 °C overnight. After incubation, the plates were washed six with 1 x TBST and were blocked with 2% BSA for 2 h at 37 °C. After incubation, the block solution was discarded. Serial 2-fold gradient dilutions of the serum, starting at 1:100, diluted in casein block were added to the wells, and the plates were incubated for 1 h at 37 °C. Next, the plates were washed and were then treated for 1 h with horseradish peroxidase (HRP) conjugated goat anti-mouse IgG (1:250; Abclonal) at 37 °C. Afterwards, plates were washed and incubated with the substrate, tetramethyl benzidine (TMB; TIANGEN), for 20 min at room temperature in the dark, followed by HCl (2 M; Solarbio) to terminate the reaction. The absorbance at 450/630 nm was recorded using an I-control Infinite 200 PRO microplate reader (TECAN). The ELISA endpoint titers were defined as the dilution of vaccinated serum, resulting in absorbance no less than 2.1-fold that of the average negative serum (1:100).

1. Pseudovirus-based neutralization assay

NT_50_ was determined by a pseudovirus-based neutralization assay, as described previously.^1^ Sera were tested for neutralizing activity against the pseudoviruses via mixing serial 3-fold diluted sample, starting at 1:30, with 650 TCID_50_ of SARS-CoV-2 Spike specific pseudovirus. After incubating for 1 hour at 37 °C, 5% CO_2_, we added Huh7 cells (200,000 cells/well) to each well. After 24-hour incubation, the luciferase substrate (Beyotime) was added to each well, and the luciferase signal was measured. Luciferase activity was then measured using an I-control Infinite 200 PRO microplate reader (TECAN). Neutralizing activity was calculated by quantification of luciferase activity in relative light units (RLU). NT_50_ was calculated using a log (inhibitor) vs. normalized response (Variable slope) non-linear regression model in GraphPad Prism 8.0 (GraphPad Software).

**Evaluation of cellular immune response**

The spleen cells of mice were isolated and analyzed by ELISpot and flow cytometry to determine cellular immune response.

1. ELISPOT assay

Cellular immune responses in the vaccinated mRNA-D2-LNP mice were assessed using IFN-γ or IL-4 precoated ELISPOT kits (MabTech), according to the manufacturer’s protocol. Briefly, the plates were four washed with PBS and blocked using RPMI 1640 (Thermo Fisher Scientific) containing 10% FBS and incubated for 30 minutes at room temperature. Immunized mouse splenocytes (300,000 cells/well) were stimulated with peptide pool for SARS-CoV-2 RBD protein (2 μg/ml of each peptide), PMA and Ionomycin (Dakewe) as positive control and RPMI 1640 media as negative control. Following incubation at 37 °C, 5% CO_2_ for 24 hours, plates were washed with PBS and incubated with biotinylated anti-mouse IFN-γ or IL-4 antibody for 2 h at room temperature. Wash the plates and incubate Streptavidin-HRP for 1 h at room temperature. Finally, the plates were incubated with TMB substrate solution until spots exposed on the plates. Wash the plates with deionized water and dry in a dark place for 24 hours. The spots were read by an automated VSR07 ELISpot reader (AID). The numbers of spot-forming cells (SFU) per million cells were calculated by vSpot 7.0 software.

1. Flow cytometry assay

Effector memory T (Tem) cell proliferation in immunized mice were analyzed by flow cytometry assay. Briefly, immunized mouse splenocytes (1,000,000 cells/well) were stimulated with peptide pool for SARS-CoV-2 RBD protein (2 μg/ml of each peptide) for 12 h in a 5% CO2 environment at 37 °C. Brefeldin A (5 μg/ml; Biolegend) was incubated with splenocytes for 4 h. Then, Fc receptors of cells were blocked using CD16/CD32 antibodies (Mouse BD Fc Block; BD Bio-sciences) for 15 min at 4 °C, and splenocytes were stained with a cocktail of fluorescently conjugated antibodies to CD3 (PE/Cyanine7; Biolegend), CD4 (FITC; Biolegend), CD8 (PercP; Biolegend), CD44 (PE; Biolegend) and CD62L (APC; Biolegend) for another 30 min at 4 °C in dark. Following washing with cell staining buffer (BD Biosciences), dead cells were stained with Fixable Viability Dye eFluor™ 780 (Thermo Fisher Scientific) for 30 min at 4 °C in dark. Final wash with cell staining buffer, data were obtained by FACS Aria II flow cytometer (BD Biosciences) and analyzed by Flow J software, The effector memory CD4^+^ or CD8^+^T-cell response were represented as CD3^+^/CD4^+^ or CD8^+^/CD44^+^/CD62L^-^.

**Quantification of viral RNA in tissues of challenged mice by RT-qPCR**

The viral RNA in lung and tracheal tissues from challenged mice was detected by quantitative reverse transcription PCR (RT-qPCR). Briefly, tissue samples were weighed and the viral RNA in the tissues was extracted using the QIAamp Viral RNA Mini Kit (QIAGEN). SARS-CoV-2 RNA quantification was performed by RT-qPCR using the One Step PrimeScript RT-PCR kit (Takara) with the SARS-CoV-2 specific primers and probes targeting the S gene of SARS-CoV-2. Viral RNA load was expressed on a log10 scale per gram of viral RNA equivalent.

***In vivo* toxicity**

To evaluate the *in vivo* toxicity of mRNA-D2-LNP vaccine, the body weights were recorded three days after vaccination. The liver and kidney functions were analyzed on Chemray 240 and Chemray 800 (Rayto) automated Biochemical Analyzers after 24 h post vaccination in mice vaccinated with mRNA-D2-LNP (20 μg; n = 3).

For histopathology, organ tissues, including heart, liver, spleen, lung and kidney, were extracted at 24 h post inoculation, and fixed in 4% neutral-buffered formaldehyde for 24 h. Afterwards, it was embedded in paraffin, sectioned and stained with hematoxylin and eosin (H&E). Images were captured by NIKON Eclipse CI microscope.

**Statistical analysis**

Statistical analyses were performed using GraphPad Prism 8.0 (GraphPad Software). All of date are presented as the mean ± SEM. Statistical difference was analyzed by one-way or two-way ANOVA. All tests are accepted as statistically significant when the *p* value is less than 0.05.

**REFFERENCES**

1. Zhang, N.N. et al. A Thermostable mRNA Vaccine against COVID-19. *Cell* **182**, 1271-1283 (2020).


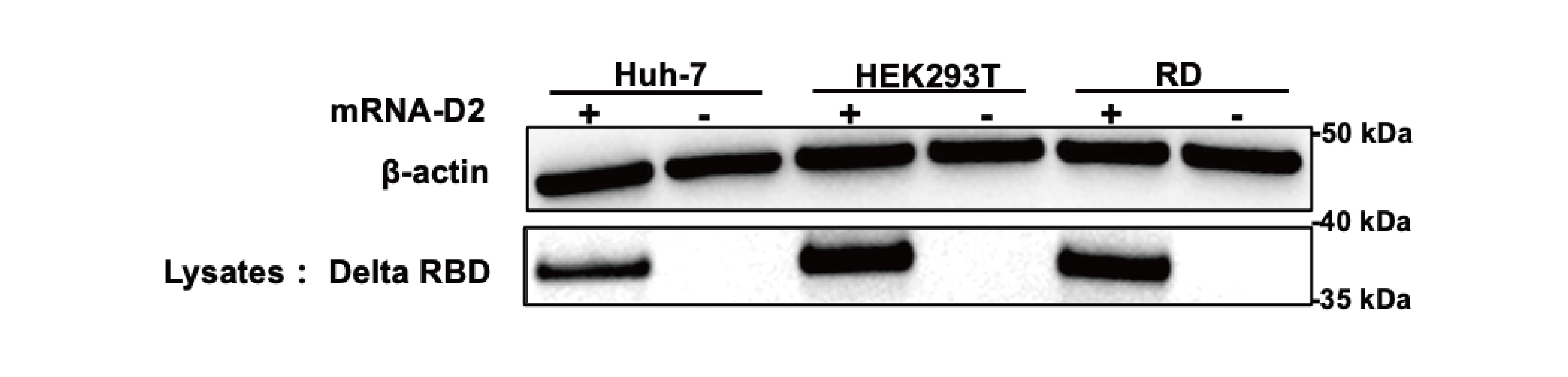


**supplementary Fig. 1. *In vitro* delivery effects were assessed by Western blotting.**

Huh7, HEK293T and RD cells were transfected with mRNA-D2-LNP (1 μg/ml) for 16 h. Lysates were collected for Western blotting to test the *in vitro* delivery of mRNA-D2-LNP.


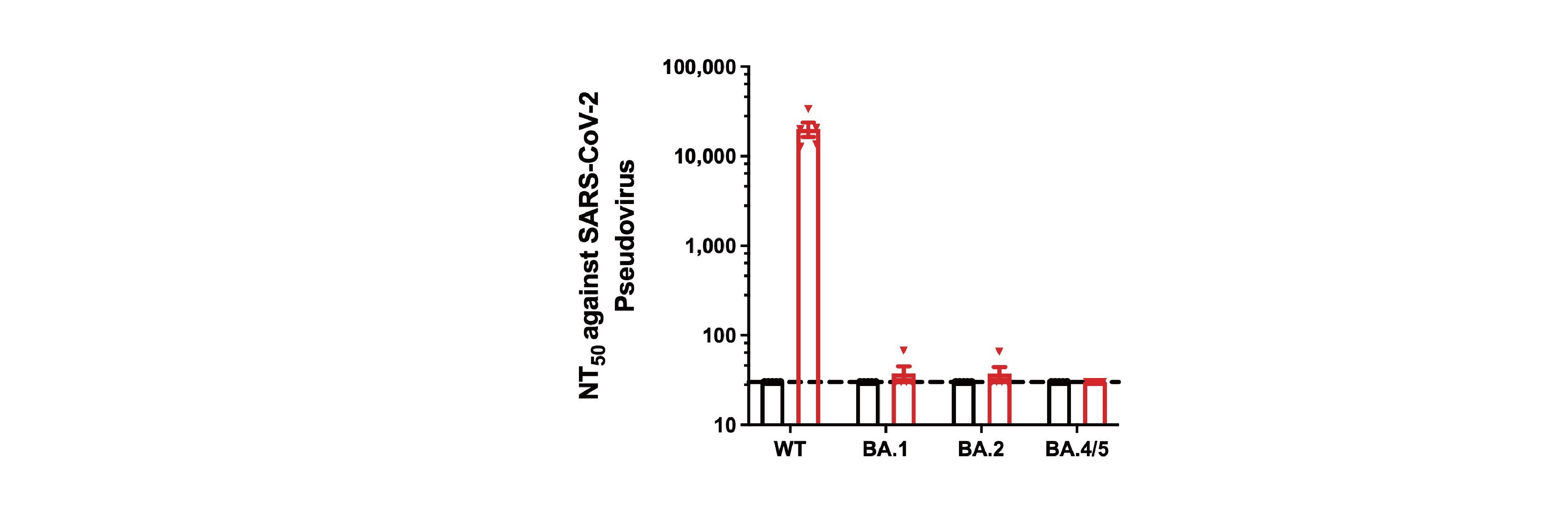


**supplementary Fig. 2. Neutralization of WT and Omicron by WT-based mRNA vaccines.**

Mice were immunized with 10 μg of WT-based mRNA vaccine and sera were collected 24 days after immunization. The sera were measured for pseudovirus-neutralizing antibody titers against WT, BA.1, BA.2 and BA.4/5. Data are shown as mean ± SEM.


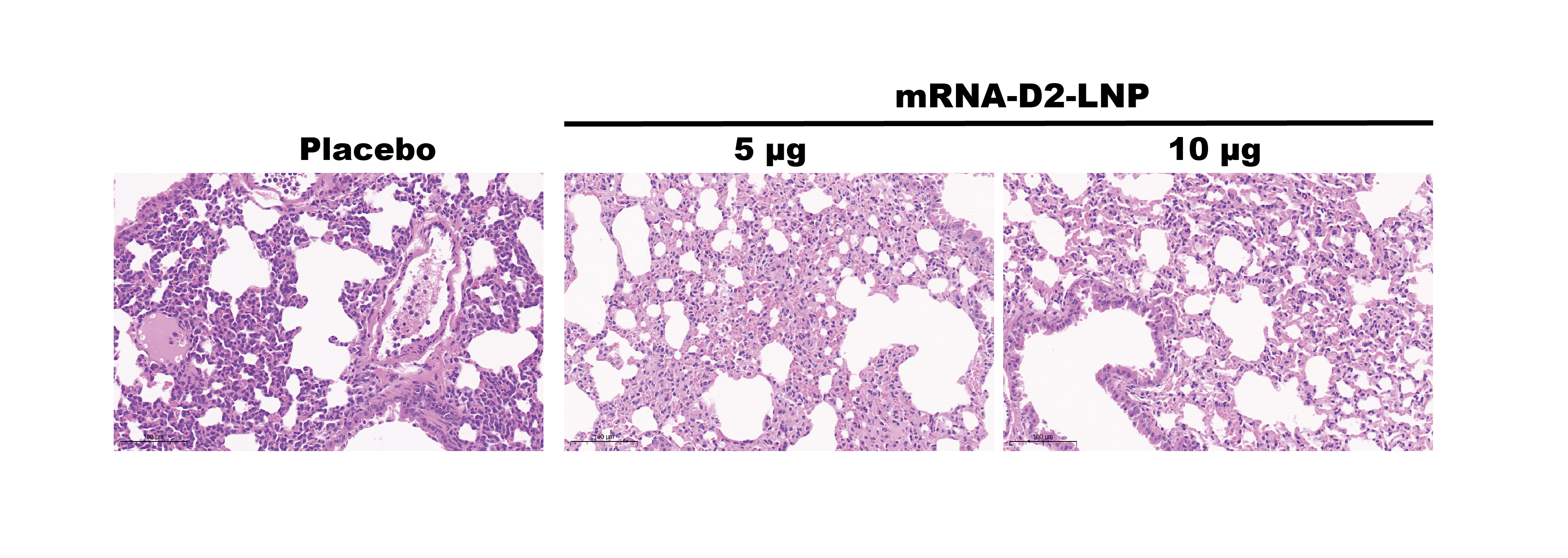


**supplementary Fig. 3. mRNA-D2-LNP protect the lungs from viral challenge.**

Mice were challenged intranasally with BA.1 (3.0×10^3^ TCID_50_) at week 7 after immunization and euthanized four days later. Lung tissue was obtained for H&E staining to assess the pathological changes. Scale bar = 100 μm, 200 ×.


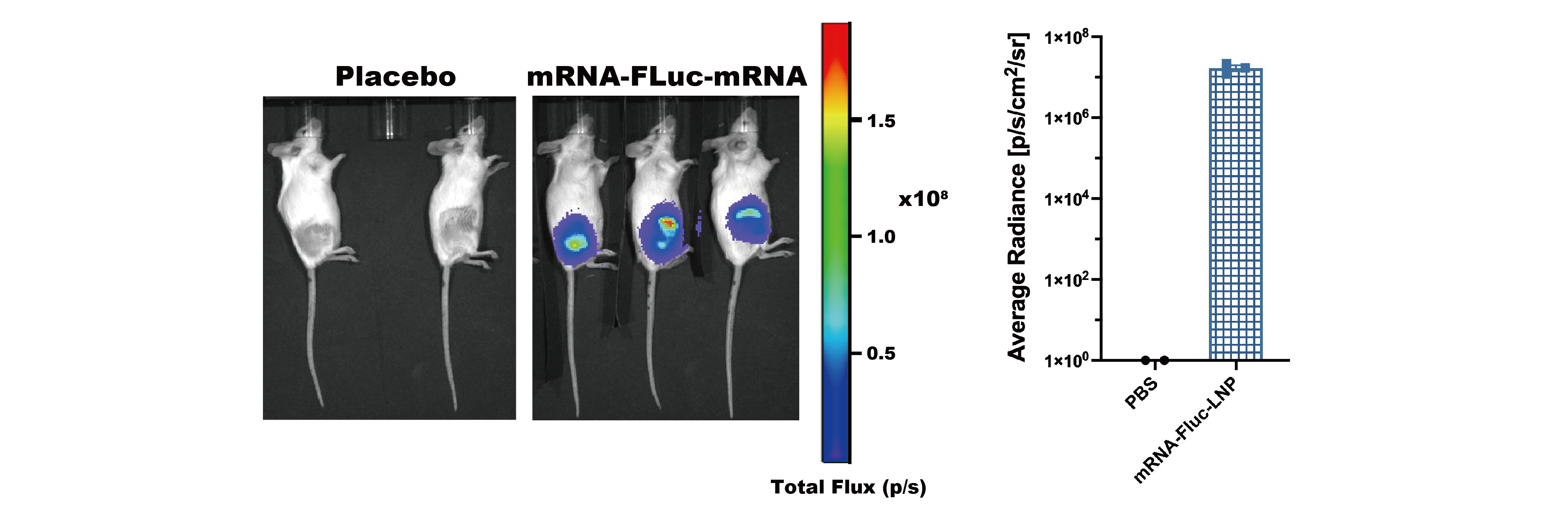


**supplementary Fig. 4. *In vivo* delivery of mRNA-LNP formulation.**

*In vivo* delivery effects were assessed by BLI. BLI to detect luciferase expression in mice 6 h after i.m. injection of mRNA-FLuc-LNP, and quantitation of the fluorescence signal in the region of interest (ROI) was conducted using Living Image 3.0.


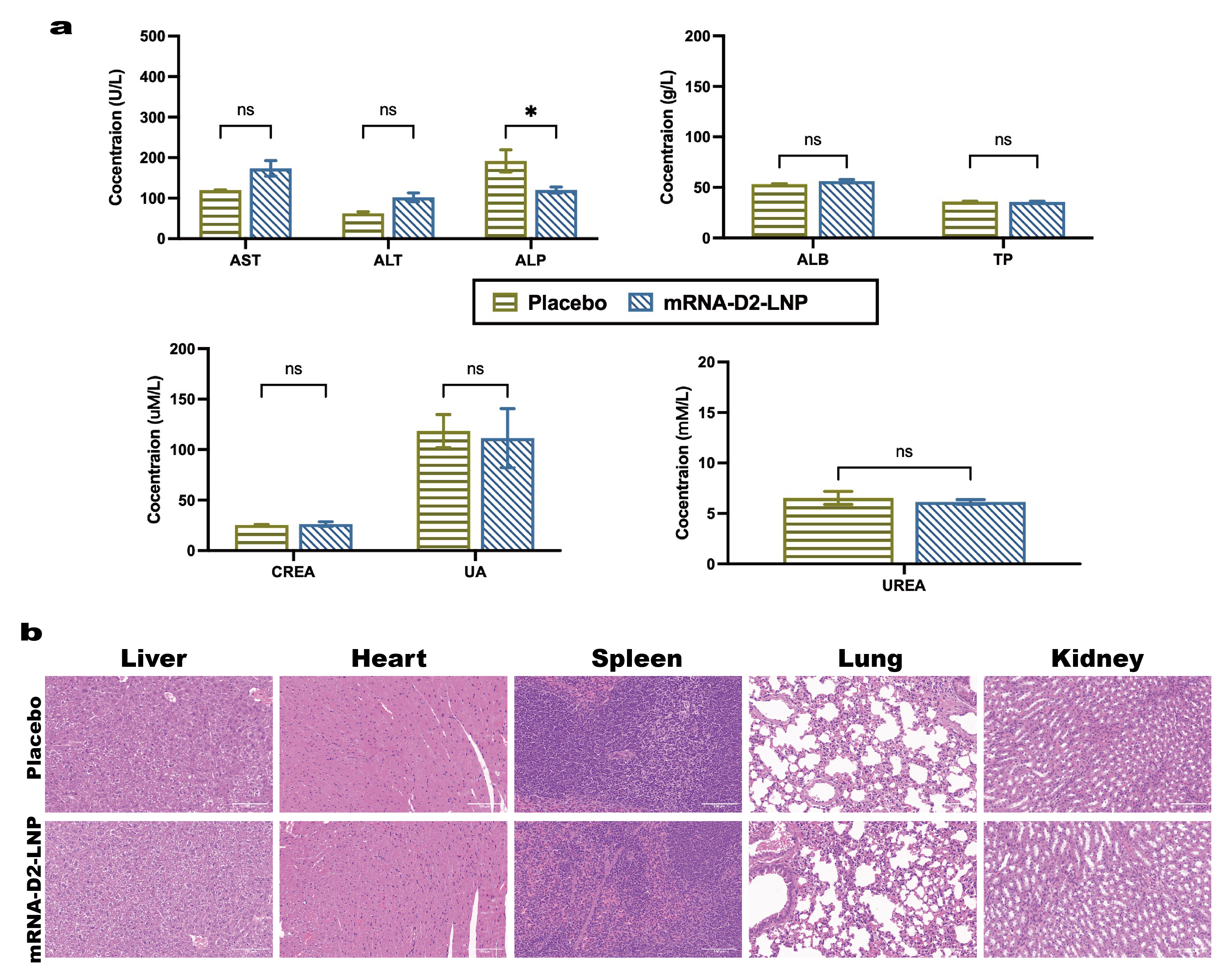


**supplementary Fig. 5. The safety evaluation of mRNA-D2-LNP in mice.**

**a** Serum was collected 24 hours after injection to determine liver and kidney function by testing blood biochemical indexes (n = 3). CREA, UA and UREA represented kidney function, while ALT, AST, ALP, TP and ALB represented the liver function. **b** Representative histopathology (H&E) of different tissues, liver, heart, spleen, lung and kidney, in mRNA-D2-LNP or DPBS group at 24 h post inoculation. Scale bar = 100 μm, 200 ×. Data are shown as mean ± SEM. Data were analyzed by two-way ANOVA with multiple comparison tests. (no significant (ns) *p* > 0.05, * *p* < 0.05)


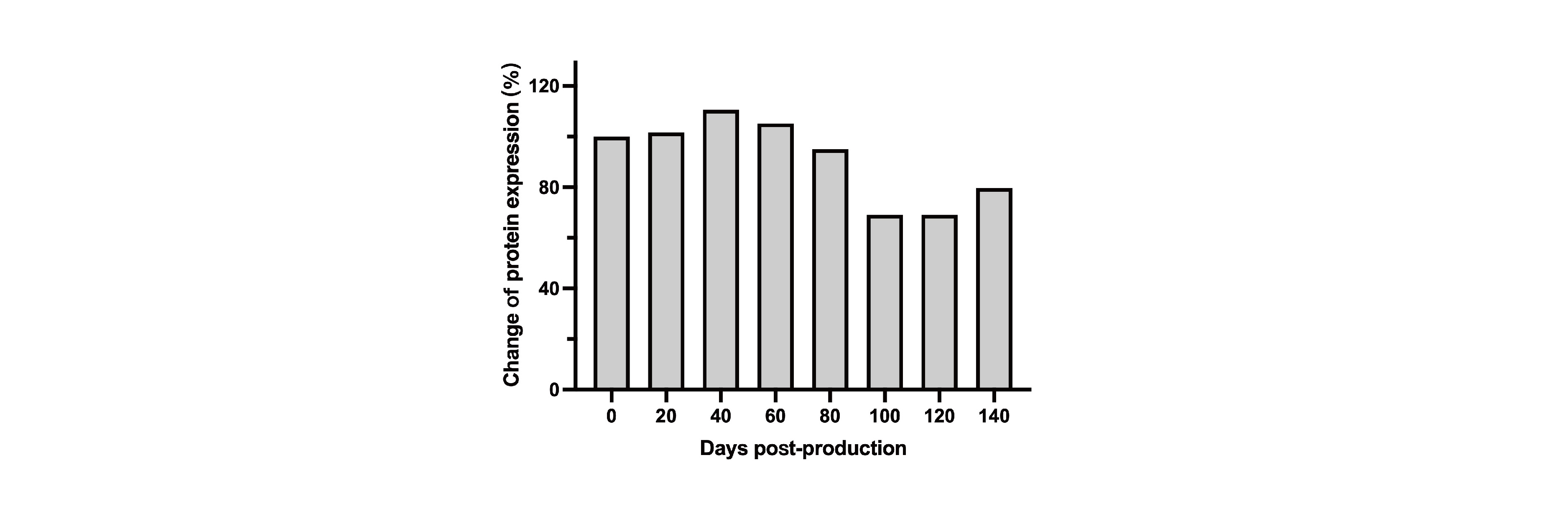


**supplementary Fig. 6. The long-term stability evaluation of mRNA-D2-LNP, Related to Figure 1p.**

Changes in Delta RBD protein expression over time were assessed by grey analysis using Image J software
